# Toxic versus Therapeutic Effects of Natural Products on Reproductive Disorders

**DOI:** 10.1155/2019/9791506

**Published:** 2019-11-18

**Authors:** Arielle Cristina Arena, Cândida Aparecida Leite Kassuya, Glaura Scantamburlo Alves Fernandes, Wellerson Rodrigo Scarano

**Affiliations:** ^1^Universidade Estadual Paulista, São Paulo, Brazil; ^2^Universidade Federal da Grande Dourados, Dourados, Brazil; ^3^Universidade Estadual de Londrina, Londrina, Brazil

The idea for this special issue emerged from increased interest in alternative therapies, especially the use of products of natural origin, for the prevention and/or treatment of reproductive disorders. However, natural products can also negatively affect male and female reproductive tracts; if the exposure occurs before and after the conception, it can affect parents as well as the offspring. Since studies focusing on the toxic versus therapeutic effects of natural products on reproductive disorders are still scarce, the purpose of this special issue is to show therapeutic effects as well detecting potential reproductive health hazards of natural products. For this special issue, researchers from different countries were invited to contribute with original research and review articles. After the review process, 12 high-quality papers were accepted for publication. The main topics addressed in this special issue include the therapeutic effects of natural products on male or female reproductive disorders, the toxicity of natural products on the male reproductive tract, and embryotoxicity and teratogenic effects of natural products. A summary of all accepted articles is provided below.

Some papers have focused on studying the benefits of natural products to treat male reproductive disorders. J. Wang and colleagues have demonstrated that Shengjing capsules (Chinese herbal medicine) could be an important therapeutic medicine for the treatment of nonobstructive azoospermia (NOA). The authors have proposed a mechanism associated with the PI3K/AKT pathway modulation to activate spermatogonial stem cells in the NOA rats. These findings provide new insights for the treatment of NOA. In another paper, Z. Zhu and colleagues have shown the beneficial effects of a water-soluble polysaccharide extracted from *Morinda officinalis* (MOP) on varicocele rats. The results of this study have demonstrated that MOP treatment improved the sperm parameters in varicocele rats through the angiogenesis inhibition in testes and a relative upregulation of a VEGF (specific mitogen of vascular endothelial cells) and MMP-9 (the primary mediators of extracellular matrix degradation).

S. M. Ezzat and colleagues have studied the mechanisms by which the *Eurycoma longifolia*, a well-recognized aphrodisiac herb, improves the erectile dysfunction, using an in vitro model. The authors have observed that the aqueous extract of *Eurycoma longifolia* inhibited the ROCK-II activity, leading to contraction of smooth muscle. These findings revealed important insights about the role of this species in male sexual disorders. Still, on the same subject, P. M. Kameni and coauthors have investigated the suppressive effect of *Nymphaea lotus* (a species used as an aphrodisiac, astringent, and anti-inflammatory) on erectile dysfunction induced by nitric oxide deficiency in rats. The results revealed that this species could be an excellent candidate for the treatment of erectile dysfunction.

In another paper, S. M. Ezzat and colleagues have studied the in vivo effects of aqueous extract of *Eurycoma longifolia* on male reproductive functions and the brain cortical and hippocampal content of dopamine, serotonin, and noradrenaline. The authors have confirmed the aphrodisiac and anabolic activities of the extract in male rats, meaning the effects were attributed to an increase of testosterone level as well as enhancement of brain cortical and hippocampal dopamine content.

The paper by J. Park and colleagues has reported that the Aconiti Lateralis Radix Preparata (AL) can be a therapeutic alternative for the treatment of benign prostatic hyperplasia (BPH). In this study, the treatment with AL ameliorated pathological proliferation in the prostate as well as decreased the two main factors associated with the BPH pathogenesis. Additionally, AL effects did not cause testicular apoptosis, a common adverse effect caused by finasteride, a reference drug used to treat BPH. These results suggest AL as a potential therapeutic agent for BPH treatment.

S. Li and colleagues presented some aspects of male reproductive toxicity of natural products. In this review, the authors have reported that several toxic compounds present in natural products could be used for therapeutic purposes. Some substances that are spermatotoxic may be useful for contraception at therapeutic dosage.

Other papers have focused on studying the therapeutic effects of natural products on female reproductive disorders. C. H. Wu and colleagues have investigated the mechanisms by which the herbal formula B401, widely used in Taiwan, may relieve the symptoms associated with menopause. The treatment with herbal formula B401 in ovariectomized mice confirmed the usefulness of this supplement for alleviating discomfort symptoms in middle-aged women. Y. Wang and coauthors have evaluated the effects of the polysaccharides of *Fructus corni* on ovarian functions in naturally aging female mice. The obtained data demonstrated that this compound improved the ovarian function in aging-associated perimenopause symptoms, indicating that this natural product can be a promising therapeutic alternative for symptoms associated with menopause.

In an in vitro study, Q. Zhou and colleagues have studied the efficacy of formononetin, an isolated ingredient from *Astragalus membranaceus*, in improving the tumoricidal effect of everolimus, an inhibitor of serine-threonine kinase mammalian target of rapamycin with broad antitumor activities. The results showed that the combination treatment of formononetin plus everolimus might be an effective approach for breast cancer chemotherapy. In another paper, X. Mao and colleagues have investigated a new alternative for the treatment of cervicitis, a common sexually transmitted disease, in a mouse model. To this end, the Feilin Vaginal Gel (FVG), a Chinese herbal formula, was tested and the obtained results demonstrated that the FVG might be an alternative for the treatment of cervicitis.

A. A. Alafiatayo and colleagues have assessed the effects of *Curcuma longa* extract, a Southeast Asia traditional medicine, on embryotoxicity and teratogenic effects in Zebrafish. The obtained findings showed that the extract has potential specific toxic effects on embryos and larvae development, especially at a higher dosage, indicating that *Curcuma longa* needs further investigation.

We hope that this special issue can be *really special* for scientists studying the effects of natural products as an alternative medicine, as well as their possible adverse effects focusing on the reproductive system.

